# 5-ALA-mediated fluorescence of musculoskeletal tumors in a chick chorio-allantoic membrane model: preclinical in vivo qualification analysis as a fluorescence-guided surgery agent in Orthopedic Oncology

**DOI:** 10.1186/s13018-022-02931-x

**Published:** 2022-01-15

**Authors:** Wiebke K. Guder, Wolfgang Hartmann, Clarissa Buhles, Maike Burdack, Maike Busch, Nicole Dünker, Jendrik Hardes, Uta Dirksen, Sebastian Bauer, Arne Streitbürger

**Affiliations:** 1 Department of Orthopedic Oncology, University Hospital Essen, Hufelandstrasse 55, 45147 Essen, Germany; 2 Division of Translational Pathology, Gerhard-Domagk-Institute of Pathology, University Hospital Muenster, Albert-Schweitzer-Campus 1, Building D17, 48149 Muenster, Germany; 3 Department of Neuroanatomy, Institute for Anatomy II, University of Duisburg Essen, University Medicine Essen, Hufelandstrasse 55, 45147 Essen, Germany; 4 Department of Pediatric Hematology and Oncology (III), University Hospital Essen, Hufelandstrasse 55, 45147 Essen, Germany; 5 Sarcoma Center, West German Cancer Center, University Hospital Essen, Hufelandstrasse 55, 45147 Essen, Germany

**Keywords:** Musculoskeletal tumor, Sarcoma, 5-Aminolevulinic acid, Chick chorio-allantoic membrane model, Tumor fluorescence, Photodynamic detection

## Abstract

**Background:**

Fluorescence-guided surgery (FGS) with 5-aminolevulinic acid (5-ALA) and other contrast agents has shown its efficacy in improving resection margins, local recurrence and survival rates in several medical disciplines. It is the objective of this study to analyze the engraftment rate of musculoskeletal tumor specimens on the chick chorio-allantoic membrane (CAM), the rate of tumor fluorescence (PDD), and the effects of photodynamic therapy (PDT) after exposure of tumors to 5-ALA in an in vivo environment.

**Methods:**

A total of 486 CAMs were inoculated with macroscopic tumor grafts (*n* = 26; *n* = 478 eggs) and primary cell culture suspensions (*n* = 2; *n* = 8 eggs) from 26 patients on day 10 of egg development. On day 16, 2 mg/200 µl 5-ALA were topically applied per egg. After 4 h of incubation, Protoporphyrin IX was excited using blue light (420 ± 10 nm). Tumor fluorescence (PDD) was photo-documented. A subgroup of specimens was additionally exposed to red light (635 nm ± 10 nm; PDT). After the termination of the experiment, CAM-grown tumors were histopathologically analyzed.

**Results:**

Benign and borderline tumors (chondroblastoma, giant cell tumor of bone and atypical chondrogenic tumor) presented with high rates of detectable fluorescence. Comparable results were found for chondrosarcoma, osteosarcoma and Ewing’s sarcoma among bone and dedifferentiated liposarcoma, myxofibrosarcoma and undifferentiated pleomorphic sarcoma among soft tissue sarcomas. Overall, tumor fluorescence was negative for 20.2%, single-positive (+) for 46.9% and double-positive (++) for 32.9% of macroscopic xenografts, and negative in 20% and (+) in 80% of primary cell culture tumors. Macroscopic tumor xenografts (*n* = 478) were identified as viable in 14.8%, partially viable in 2.9% and partially to completely regressive in 45.2%. All (*n* = 8) tumors grown from primary cell culture were viable. After PDT, tumor samples were found viable in 5.5%, partially viable in 5.5% and partially to completely regressive in 68%. Egg survival increased with decreasing PDT doses.

**Conclusions:**

The CAM model proves to be a suitable in vivo model for the investigation of short-term observation questions in musculoskeletal tumors. The findings of this study warrant further investigation of PDT effects on musculoskeletal tumors and a possible incorporation of 5-ALA FGS in clinical Orthopedic Oncology care.

## Background

Fluorescence-guided surgery (FGS) with contrast agents such as indocyanine green (ICG), 5-aminilevulinic acid (5-ALA) and methylene blue (MB) has been introduced into a number of medical disciplines with an intention of detecting sentinel lymph nodes and improving resection margins, local recurrence and survival rates [1–3]. In dermatology, photodynamic therapy (PDT) has been established as a highly effective treatment for a variety of skin conditions, including pre-cancerous lesions, superficial non-melanoma skin cancers, or inflammatory acne vulgaris [4]. The evaluation of contrast agents in the treatment of musculoskeletal tumors has also become a subject of preclinical studies in the recent past [5]. Kusuzaki et al. reported that tumor fluorescence could be detected in lesions measuring as small as 1 mm using acridine orange (AO) in a mouse osteosarcoma model [6]. The qualification of 5-ALA in photodynamic detection (PDD) and photodynamic therapy (PDT) of musculoskeletal sarcoma has also been investigated with promising results in in-vitro studies [7, 8].

Intralesional curettage of giant cell tumors of bone (GCTB) or atypical chondrogenic tumors (ACT) of the appendicular skeleton represents the current treatment recommendation for these locally aggressive musculoskeletal tumors. Compared with extralesional resections, they are associated with an increased rate of local recurrences, which are reported to be as high as 10–40% in the literature [9–11]. In addition, local recurrences after soft tissue or bone sarcoma resections occur in the soft tissues directly adjacent to the primary tumor site, even when the pathological examination of the resection specimen concluded a resection with clear margins (R0). Residual microscopic tumor and discontinuous tumor growth, possibly also alongside tumor-feeding vessels, are a likely cause for these local recurrences. In addition, a reduction of tumor size due to neoadjuvant chemotherapy or radiation treatment may further increase the risk of leftover discontinuous microscopic tumor lesions outside the tumor capsule [12, 13].

Therefore, contrast-agent assisted curettage and visualization of tumor fluorescence (PDD) for tumors treated by intralesional curettage seems a promising tool in reducing local recurrence rates and the necessity of repeated operations. FGS-assisted evaluation of both the tumor bed and margins of the resection specimen after marginal or wide resection may be able to detect discontinuous tumor growth—that would otherwise have gone unnoticed by both surgeon and pathologist—and enable targeted re-resections of affected or suspicious tissues. Furthermore, PDT treatments may also be able to devitalize remaining microscopic tumor regardless of resection technique.

Against this background, it is the objective of this study to assess the aptitude of 5-ALA as a fluorescence-guided surgery agent for musculoskeletal tumors in a chorion-allantois membrane (CAM) model. 5-ALA was chosen as the investigated contrast agent given its known effect on the heme metabolism and a lack of severe reported side effects [2]. The CAM model is suitable as an in vivo patient-derived xenograft (PDX) model [14]. Reported successful engraftment of fresh solid tumor aliquots, preservation of their associated matrix as well as the microenvironment of heterogeneous tumor cell populations made us select the CAM model for this study [15]. The chick embryo’s immune deficiency and the model’s low cost, short investigation period, easy reproducibility, and reliability were other contributing factors [16, 17]. The main research questions were: (1) whether and at what rate musculoskeletal tumor xenografts engraft on the CAM; (2) whether detection of tumor fluorescence is successful in an in vivo situation against the CAM background; (3) which PDT doses are tolerated by the chick embryo and (4) whether a therapeutic effect of PDT can be determined.

## Material and methods

### Patient characteristics

Twenty-six patients with musculoskeletal tumors were included in this study. Pre-operative workup included plain radiographs and/or computed tomography (CT) scans (for bone tumors) and magnetic resonance imaging (MRI) studies (for all patients). Primary, locally recurrent and metastatic musculoskeletal tumors regardless of prior neoadjuvant treatments were considered eligible for inclusion. Patient characteristics, pre-treatment information, histological diagnosis, tumor grading, and the amount of remaining viable tumor, if applicable, were recorded (see Table 1).

**Table 1:** Patient, tumor and CAM characteristics

Patient/tumor data									CAM data					
#	Age	Sex	Diagnosis	Grade	Tumor	OP	Pre-treatment	Viable tumor	Inoculated eggs (*n*)	Tumor sample/egg (*n*)	Evaluable eggs (*n*)	Confirmed histology (*n*)	5-ALA fluoresc. (*n*)	Contaminated eggs (*n*)
*Bone tumors—fresh tumor aliquots*														
1	37	F	Ganglion cyst	–	Primary	Curr	–	–	10	3	8	8	Neg	0
2	14	F	Chondroblastoma	–	Primary	Curr	–	–	5	1	5	4	Pos (++)	0
3	30	F	GCTB	–	Primary	Curr	–	–	21	2	17	17	Pos (+/++)	0
4	50	F	ACT	1	Primary	Curr	–	–	10	1	9	9	Pos (+/++)	0
5	47	M	Chondrosarcoma	2	Primary	Res	–	–	29	3	12	6	Pos (+)	5 (excl.)
6	66	F	Chondrosarcoma	2–3	Primary	Res	–	–	6	2	5	5	Pos (+/++)	0
7	52	F	Chondrosarcoma	3	Primary	Res	–	–	26	13:4; 13:1	18	7	Pos (+/++)	0
8	37	F	Osteosarcoma	3	Primary	Res	Chemo	90%	8	2	7	6	Pos (+/++)	0
9	10	F	Osteosarcoma	3	Primary	Res	Chemo	10%	13	3	4	4	Pos (+)	0
10	54	F	UPS	3	Primary	Res	Chemo	40%	15	3	15	14	Pos (+)	0
11	59	M	NOS	3	Primary	Res	Chemo	80%	26	2	23	20	Pos (+/++)	0
12	28	M	Ewing sarcoma	3	Meta. Met	Biopsy	–	–	28	2	23	7	Pos (+/++)	2 (excl.)
13*	28	M	Chordoma	3	Meta. Met	Biopsy	–	–	15	2	10	1	Neg	0
*Soft tissue tumors—fresh tumor aliquots*														
1	57	M	Vascularized soft tissue	–	Primary	Res	–	–	5	1	3	3	Neg	0
2*	27	F	PVNS	–	Primary	Biopsy	–	–	18	3	14	9	Pos (+)	1 (excl.)
3*	27	F	PVNS	–	Primary	Res	–	–	13	4	11	9	Pos (+)	1 (excl.)
4	19	F	EMC	–	Primary	Res	Chemo	80%	14	2	10	7	Pos (+)	0
5	42	M	Myxoid liposarcoma	1	Meta. Met	Biopsy	–	–	23	1	14	4	Neg/pos (+)	2 (excl.)
6	74	M	Dediff. liposarcoma	2	Primary	Res	–	–	33	2	20	18	Pos (+/++)	6 (excl.)
7	75	M	Dediff. liposarcoma	2	Primary	Res	–	–	25	1	14	12	Pos (++)	0
8	78	M	Myxofibrosarcoma	3	Primary	Res	–	–	32	2	26	12	Pos (++)	1 (excl.)
9	71	M	Myxofibrosarcoma	3	Recurrence	Res	–	–	27	2	19	14	Pos (+/++)	1 (excl.)
10	73	M	UPS	3	Primary	Res	–	–	30	1	19	12	Pos (+/++)	0
11	81	M	UPS	3	Primary	Res	–	–	18	3	13	13	Pos (++)	1 (excl.)
12	79	F	UPS	3	Primary	Res	–	–	15	2	12	11	Neg/pos (+)	0
13	74	F	UPS	3	Primary	Res	ILP	20%	13	4	8	6	Pos (+/++)	0
*Bone tumors—primary cell culture*														
1*	29	M	Chordoma	3	Meta. Met	Res	–	–	4	1	2	2	Pos (+)	0
2	75	M	Dediff. chondrosarcoma	3	Primary	Biopsy	–	–	4	1	4	4	Pos (+)	0

#, number; OP, operation; (*n*), number; fluoresc., fluorescence; *, same patient; F, female; M, male; GCTB, giant cell tumor of bone; ACT, atypical chondrogenic tumor; UPS, undifferentiated pleomorphic sarcoma; NOS, sarcoma not otherwise specified; PVNS, pigmented villo-nodular synovitis; EMC, extraskeletal myxoid chondrosarcoma; Meta., Met metachronous metastasis; Curr, curettage; Res, resection; chemo, chemotherapy; ILP, isolated limb perfusion; neg, negative; pos, positive; excl., excluded

### Tumor tissue aliquot preparation for inoculation with fresh tumor samples

Given that sufficient representative tissue was available (*n* = 26), an experienced pathologist separated a fresh tumor tissue sample from biopsy, curettage, or resection specimens without impairing standard diagnostic procedures, documenting the macroscopic aspect of the lesional tissue to be inoculated on the CAM and in the diagnostic specimen. Biopsy aliquots were minced in a petri dish using a scalpel under laminar airflow. For primary cell culture, specimens were further processed as follows: 1–2 ml DMEM was added and the biopsy tissue was strained through a sieve (Corning^®^ Costar^®^ Cell Strainer 100 µm). The suspension was centrifuged for 3 min at 800 revolutions per minute (RPM). Then, the supernatant was pipetted into a T75 tissue culture flask. The remaining pellet was digested for 15 min at 5% CO_2_ and 37 °C using 5–7 ml trypsin (trypsin EDTA, 0.25%). Tissue digestion was inhibited by adding 5–7 ml DMEM, and the suspension was centrifuged again as mentioned above. The supernatant was removed, the pellet solved in DMEM, and then, transferred into a T25 tissue culture flask [18].

### Primary cell culture

Primary cell lines were grown in DMEM (Gibco^™^ Dulbecco’s Modified Eagle Medium, high glucose, GlutaMAX^™^) supplemented with 10% fetal bovine serum (FBS), 1% l-glutamine and 1% penicillin/streptomycin on T25, T75, or T175 tissue culture flasks in a humidified atmosphere of 5% CO_2_ at 37 °C. Tissue culture flasks were coated in poly-l-lysine and washed with DPBS (Gibco^™^ Dulbecco’s phosphate-buffered saline) in preparation of tumor-derived single-cell suspensions. During incubation, the vitality of primary cell cultures was checked on a daily basis under a light microscope. Cell culture medium was exchanged and the cell lines expanded depending on cell adherence, cell count and the quality of culture medium [18].

### Impact of 5-aminolevulinic acid hydrochloride (5-ALA) on heme biosynthesis, photodynamic detection (PDD) and therapy (PDT)

5-ALA is a precursor molecule in hemoglobin biosynthesis, which takes place in almost all cells of the human body. Exogenous addition of 5-ALA induces synthesis and accumulation of the intermediate substance protoporphyrin IX (PPIX), which is fluorescent and acts as a photosensitizer. Formation of heme by incorporation of iron in PPIX is catalyzed by the enzyme ferrochelatase, which has a lower activity in tumor tissues and leads to increased concentrations of PPIX [19]. Excitation of PPIX with blue light (375–440 nm) leads to the emission of red light (PDD), using a long-pass filter (440 nm). When PPIX is excited using red light (635 nm), the combination of 5-ALA, oxygen and red light leads to the generation of highly reactive oxygen species, followed by selective cell death [20]. 5-ALA used in this study was prepared at the hospital’s pharmacy. The light source (MultiLite^®^, German Medical Engineering (GME)) used in this experiment was programmed to emit either blue (420 ± 10 nm; intensity 9 mW/cm^2^) or red light (635 nm ± 10 nm; intensity 50 mW/cm^2^).

### CAM model, photodynamic detection (PDD) and therapy (PDT)

**Fig. 1:**
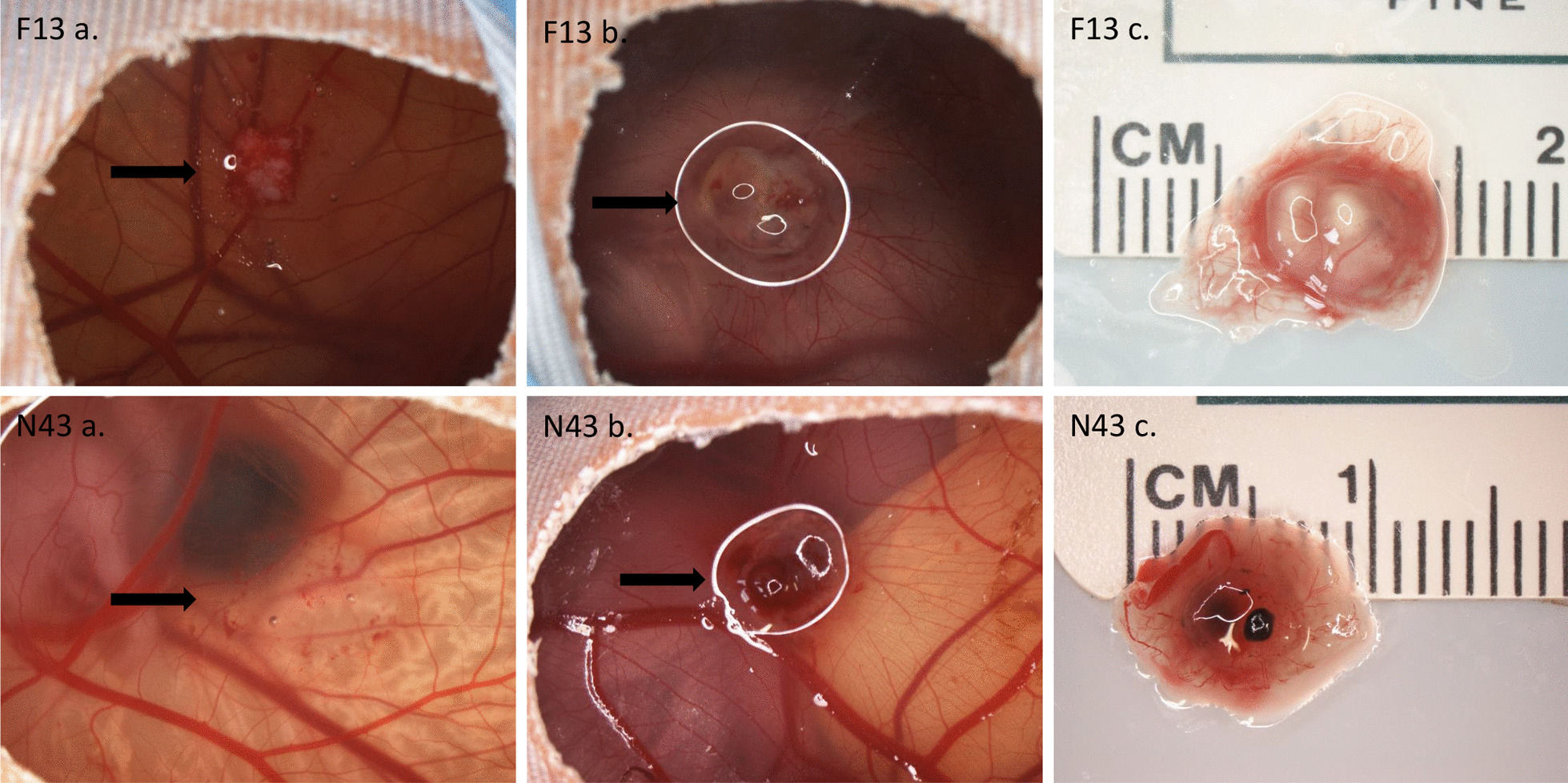
CAM and Specimen Photo Documentation (F 13—atypical chondrogenic tumor and N43—dedifferentiated chondrosarcoma). **a** CAM membrane after application of xenograft (F13) or tumor cell suspension (N43) containing 10^6^ cells—day 10 of egg development (see black arrows). **b** CAM membrane on day 16 immediately before egg termination (PDD-only group, see black arrows). **c** Scaled excised tumor xenograft (area of interest)

A CAM protocol as proposed by Sys et al. was adapted to our study design [13]. Fertilized eggs from a commercial hatchery (breed Lohmann Braun; Hof Brinkschulte GmbH & Co. KG poultry farm, Senden, Germany) were incubated in a sideways position at 37.8 °C and a humidity of 60% (ProCon automatic systems GmbH & Co. KG, Grumbach, BSS 300). On day 3 of egg development, a 1–2 mm hole was poked into the shallow part of the egg, and 3 ml of egg white was withdrawn to separate the CAM membrane from the eggshell. The hole was then sealed using adhesive tape. Afterwards, a window of approximately 2 cm in diameter was cut into the eggshell. The egg white was returned into the egg and the window was sealed by adhesive tape. Eggs without an embryo were discarded. Egg vitality was checked every second day and devitalized eggs were removed from the incubator. On day 10 of egg development, the CAM membrane was carefully incised using a scalpel, and tumor tissue aliquots were applied on the incised areas (Fig. 1: F13 a, N43 a). The number of xenografts (2–3 mm in diameter) varied depending on the amount of available tumor. After the xenograft application, the CAM was photo-documented (Nikon, SMZ1000). On day 16 of egg development, 5-ALA was dissolved in water to produce a stock solution of 2 mg/200 µl. A mean of 1–2 eggs/tumor entity were left untreated to serve as controls. The remaining eggs were topically incubated with 200 µl of 5-ALA stock solution per egg. After an incubation period of 4 h, excitation of PPIX and visualization of the resulting fluorescence (PDD) was implemented using a light source (MultiLite^®^, German Medical Engineering (GME)) emitting blue light (420 ± 10 nm; intensity 9 mW/cm^2^) at a distance of 15 cm. A long-pass filter (Noir Laser, model #50, Milford, MI, USA; optical density 190–400 nm 5+; > 400–60 nm 2+) for the emitted light was used for photo documentation, ensuring a good discrimination between tumor and healthy CAM/embryo tissue. Eggs with fluorescent tumors were then subdivided into two groups. The PDD-only group was photo-documented (Nikon, SMZ1000, Fig. 1: F13 b and N43 b) and terminated. For termination, eggs were positioned on a bed of ice for 20 min. The eggshells were then cut in half and embryos were sacrificed. Finally, the area of interest on the CAM was excised (Fig. 1: F13 c, N43 c) and processed for histopathological examination. The PDT group was treated by additional exposure to a light source (MultiLite^®^, German Medical Engineering (GME)) emitting red light (635 nm ± 10 nm; intensity 50 mW/cm^2^; 10/20/30 J/cm^2^) at a distance of 15 cm and incubated for another 24 h. On day 17, the PDT group was terminated and processed after photo documentation (Nikon, SMZ1000) using the same protocol.

### Criteria for evaluating PDD results

**Fig. 2:**
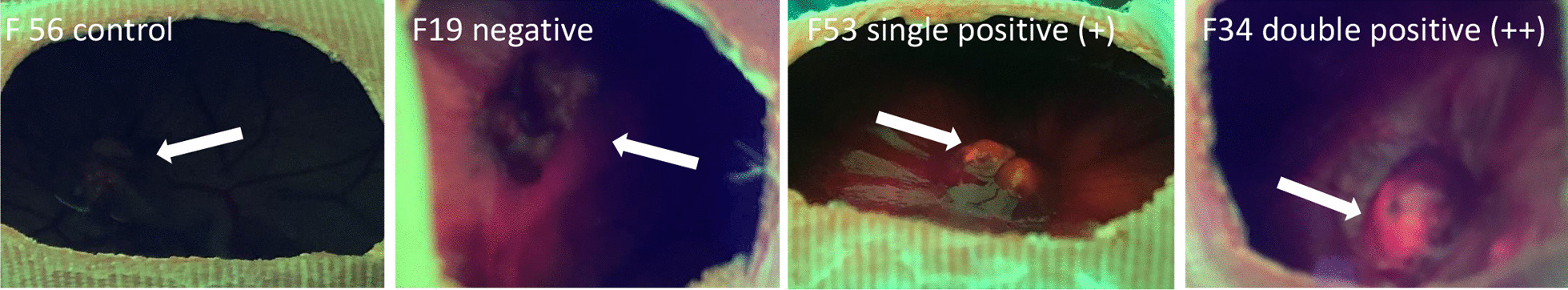
PDD evaluation (giant cell tumor of bone). F56—control, F19—negative, F53—single positive, F34—double positive

Tumor fluorescence after exposure to 5-ALA was classified as negative, single-positive (+) and double-positive (++). Control specimen were not treated with 5-ALA. In these specimens, neither the CAM background nor the tumor was fluorescent (Fig. 2, F56). In negative specimens, the tumor did not emit fluorescence, while the CAM background was fluorescent (Fig. 2, F19). Single positive fluorescence was defined as fluorescence of intermediate and/or patchy brightness (Fig. 2, F53). Double positive fluorescence was defined as fluorescence of high and/or uniform brightness (Fig. 2, F34).

### Histological analysis and criteria for microscopic evaluation of viability/regression of tumor specimens

**Fig. 3:**
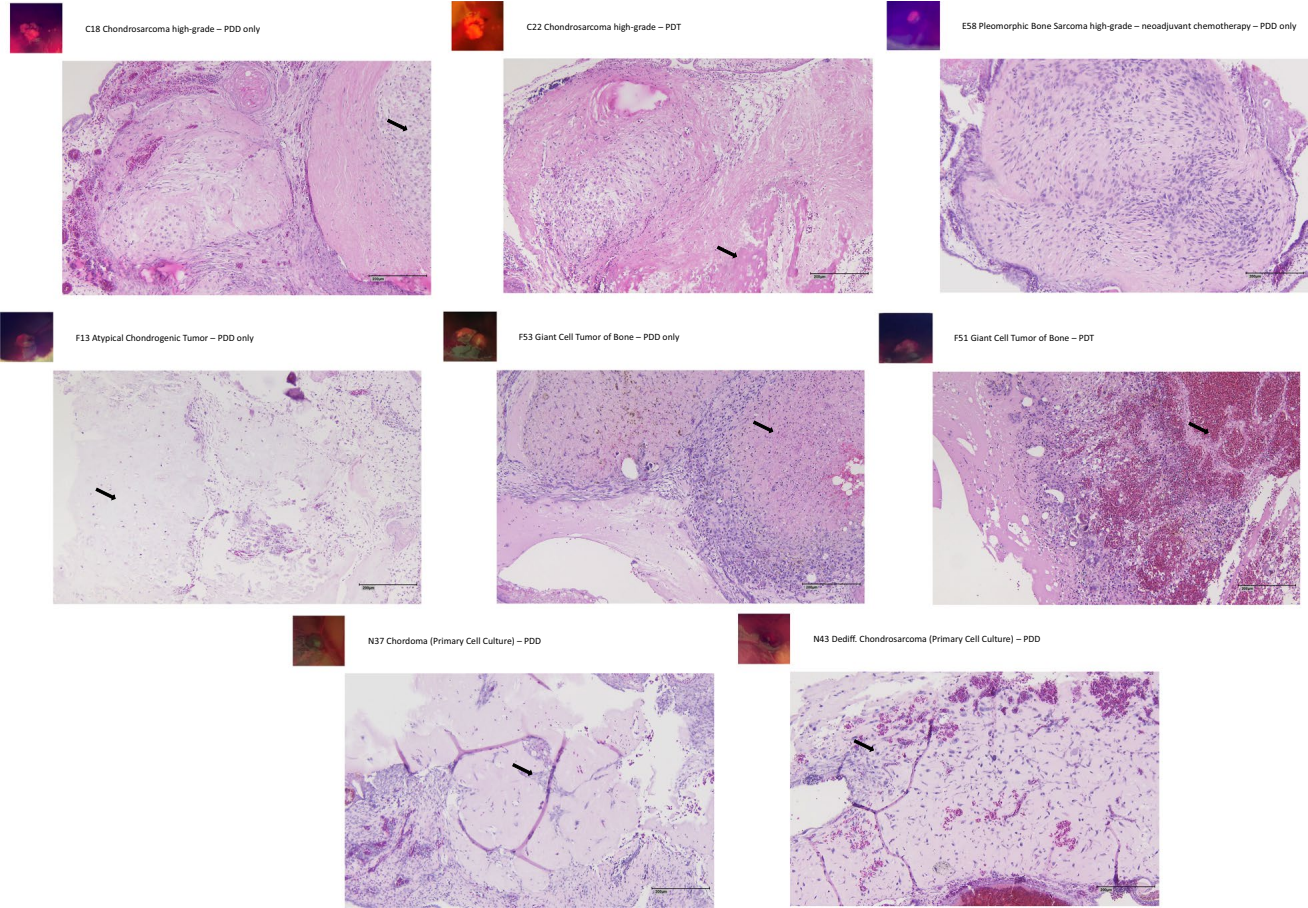
PDD/PDT and histopathological results—bone tumors. C18—chondrosarcoma high-grade. PDD only. Fluorescence +. Arrow highlights vital tumor areas in a specimen classified as viable. C22—chondrosarcoma high-grade. PDT. Fluorescence ++. Arrow highlights areas with regressive changes in a specimen classified as partially regressive. E58—pleomorphic bone sarcoma high-grade. PDD only. Fluorescence ++. Predominantly vital tumor areas in a specimen classified as viable. F13—atypical chondrogenic tumor. PDD only. Fluorescence ++. Arrow highlights residual vital tumor areas in a specimen classified as regressive. F53—Giant cell tumor of bone. PDD only. Fluorescence ++. Arrow highlights areas with necrosis and regressive changes in a specimen classified as regressive. F51—giant cell tumor of bone. PDT. Fluorescence ++. Arrow highlights areas with hemorrhage and regressive changes in a specimen classified as regressive. N37**—**chordoma (primary cell culture). PDD only. Fluorescence negative. Arrow highlights vital tumor clusters (in matrigel) in a specimen classified as viable. N43—dedifferentiated chondrosarcoma (primary cell culture). PDD only. Fluorescence +. Arrow highlights vital tumor cell with an atypical mitosis (in matrigel) in a specimen classified as viable

**Fig. 4:**
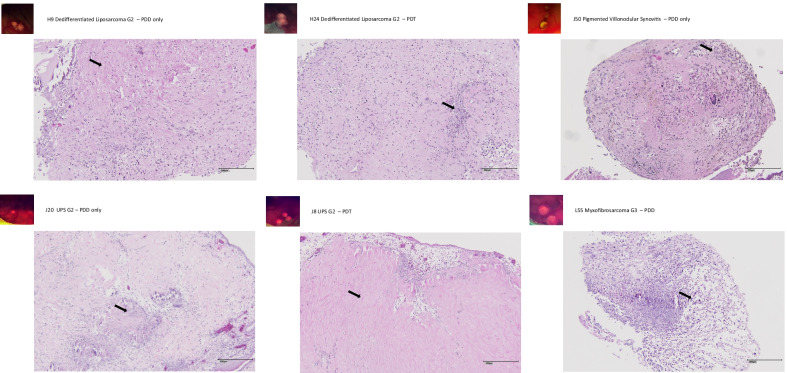
PDD/PDT and histopathological results—soft tissue tumors. H9—dedifferentiated liposarcoma G2. PDD only. Fluorescence +. Arrow highlights areas with regressive changes in a specimen classified as regressive considering the entire sample. H24—dedifferentiated liposarcoma G2. PDT. Fluorescence ++. Arrow highlights areas with regressive changes in a specimen classified as regressive considering the entire sample. J50—pigmented villonodular synovitis/tenosynovial giant cell tumor. PDD only. Fluorescence +. Arrow highlights vital tumor areas in a specimen classified as viable considering the entire sample. J20—UPS G2. PDD only. Fluorescence ++. As an example of intralesional heterogeneity, arrow highlights areas with regressive changes in a specimen classified as viable considering the entire sample. J8—UPS G2. PDT. Fluorescence ++. Arrow highlights areas with regressive changes in a specimen classified as partially regressive considering the entire sample. L55—myxofibrosarcoma G3. PDD only. Fluorescence ++. As an example of intralesional heterogeneity, arrow highlights areas with residual vital tumor in a specimen classified as regressive considering the entire sample

Tissue workup followed standard routines including fixation of the tumors in 3.7% buffered formalin for 24 h, standard dehydration and paraffin embedding. Hematoxylin and eosin (H&E) staining was performed on 3 µM tissue sections following standard protocols. Tumor viability and regression were assessed through light microscopic evaluation of the entire CAM sample, taking into account the relative amount of vital tumor tissue and necrotic or fibrotic areas, respectively. A semiquantitative assessment was made with respect to the proportions of these features, classifying tumors with > 75% vital tumor tissue as viable, > 50% vital tumor tissue as partially viable, < 50% vital tumor tissue as partially regressive, and < 25% vital tumor tissue as regressive. Figures 3 and 4 show examples of representative cases, deliberately featuring intralesional heterogeneity.

## Results

### Chorio-allantoic membrane (CAM) model

A total of 486 eggs were inoculated with musculoskeletal tumor on day 10 of egg development. Of these, 141 (29.01%) eggs were devitalized before the termination of the experiment. 478 eggs were inoculated with fresh tumor samples (139 devitalized eggs; 29.07%), eight eggs were inoculated with tumor cell suspension from primary cell culture (2 devitalized eggs; 25%).

During the incubation period, macroscopic tumor grafts did not increase in size regardless of tumor entity. Xenograft appearance, however, was more rounded at the time of egg termination (Fig. 1b, F13) compared with sharper features at the time of xenograft application (Fig. 1a, F13). After application of transparent primary tumor cell suspensions (*n* = 8), growth of solid tumors with a mean of 5.5 mm in the longest diameter was observed (range 4–8 mm (*n* = 6); chondrosarcoma mean 6.25 mm (*n* = 4); chordoma mean 4 mm (*n* = 2)) (Fig. 1 N43 a–b).

### Protoporphyrin IX fluorescence/photodynamic diagnostics (PDD)

Tumor-associated fluorescence after exposure to 5-ALA was observed for benign, borderline and malignant tumors. A detailed overview of findings is presented in Tables 1 and 2.

**Table 2:** 5-ALA associated fluorescence (PDD) and histopathological results

	5-ALA associated fluorescence (PDD)				Histology		
	Negative	+ (*n* =)	++ (*n* =)	All (*n* =)	Control (*n* =)	Contaminated (*n* =)	PDT (*n* =)
*Histology*							
Control	41	–	–	**41**			
Viable	4	26	21	**51**	8	1	4
Partially viable	1	1	6	**8**	2	–	4
Regressive	21	76	43	**140**	16	13	49
No tumor	32	31	24	**87**	15	6	15
Contaminated	1	7	10	**18**			
All (*n* =)	**100**	**141**	**104**	**345**	**41**	**20**	**72**

Bold type in Table 2 resembles sums (by row and column)

+, single positive; ++, double positive

While some tumors had a uniform fluorescence of the entire xenograft, others displayed patchy fluorescence. Figures 3 and 4 depict tumor fluorescence and corresponding histopathological findings for a selection of included tumors.

5-ALA associated fluorescence in viable macroscopic tumor specimens (*n* = 51) was negative in four (7.8%; *n* = 4/51), positive (+; Fig. 2 F53) in 26 (50.9%; *n* = 26/51), and strongly positive (++; Fig. 2 F34) in 21 (41.2%). For partially regressive tumors, those rates were negative in 12.5% (*n* = 1/8), (+) 12.5% (*n* = 1/8) and (++) 75% (*n* = 6/8). 15% (*n* = 21/140) of partially or completely regressive tumors were negative, single positive in 54.3% (*n* = 76/140) and double positive in 30.7% (*n* = 43/140). Specimens without detection of tumor tissue were negative in 36.8% (*n* = 32/87), (+) in 35.6% (*n* = 31/87) and (++) in 27.6% (*n* = 24/87).

Control tumor specimens left untreated were negative (Fig. 2 F56) for tumor fluorescence in all cases (*n* = 41; 100%). Contaminated specimens displayed fluorescence in 17 samples (85%; *n* = 17/20; including controls *n* = 2; (+) *n* = 7, (++) *n* = 10).

In sum, 20.2% of macroscopic tumor aliquots were negative (*n* = 58/286); 46.9% (+) (*n* = 134/286) and 32.9% (++) (*n* = 94/286), excluding controls (*n* = 41) and contaminated specimens (*n* = 18).

Primary cell culture specimens (*n* = 6; control *n* = 1) were negative in one (20%; *n* = 1/5) and (+) in four (80%; *n* = 4/5).

### Histological analysis

Histological analysis confirmed viable inoculated tumor xenografts in 51 samples (14.8%; *n* = 51/345; controls *n* = 8). Partial viability (> 50% of vital tumor tissue) was confirmed in 10 (2.9%%; *n* = 10/345; controls *n* = 2), while partial to complete regression (< 50% and < 25% vital tumor tissue, respectively) was observed in 156 (45.2%; *n* = 156/345; controls *n* = 16) samples. Tumor xenografts were absent in 87 samples (25.2%; *n* = 87/345; controls *n* = 15). Transplanted tissue aliquots consisted of peritumoral bone or soft tissues in those samples. Twenty (5.8%; *n* = 20/345; controls *n* = 2) samples showed signs of microbial or fungal contamination and were excluded from PDD and PDT analysis for that reason. All primary cell culture specimens were viable (100%; *n* = 6/6; controls *n* = 1) in histological examination.

### Histopathological effects of photodynamic therapy (PDT)

Seventy-two eggs were treated with PDT. Histological analysis found viable tumor samples in four (5.5%; *n* = 4/72), partially viable tumors in four (5.5%; *n* = 4/72), partially and completely regressive tumors in 49 (68%; *n* = 49/72) and an absence of residual tumor in 15 (20.8%; *n* = 15/72) samples.

Thirty-four tumors were treated with 30 J/cm^2^, nine with 20 J/cm^2^, ten with 15 J/cm^2^ and nineteen with 10 J/cm^2^. Egg survival increased with decreasing PDT doses: 30 J/cm^2^ led to egg death in eleven, 20 J/cm^2^ in seven, 15 J/cm^2^ in seven and 10 J/cm^2^ in one case each.

A meaningful interpretation of PDT-associated histological tumor necrosis was not possible due to a high rate of spontaneous tumor regression, most likely caused by insufficient perfusion of tumor areas not directly adjacent to the CAM. Even so, a visible deterioration of CAM vessels directly after completion of PDT treatment suggests an impact of PDT on the CAM and tumor perfusion.

## Discussion

In this study, 5-ALA associated fluorescence of tumor xenografts inoculated on the CAM was observed in 71% of musculoskeletal tumor specimens (*n* = 245/345). Auto fluorescence of tumor and chick tissues in the absence of topically administered 5-ALA was excluded in all specimens. False-positive fluorescence due to bacterial or fungal contamination was rare at 0.5% (*n* = 17/345) and histologically proven at a rate of 0.6% (*n* = 20/345). Tumor fluorescence was observed even in regressive tumors in 85% of the specimens (*n* = 119/140) and in 55 samples (15.9%; *n* = 55/345) without histological identification of vital tumor tissue. As multiple xenografts were grown per tumor and as fluorescence was documented using the peak result, histologically analyzed sections probably did not document the most fluorescent tumor specimen in these samples.

Among benign and borderline tumors chondroblastoma, GCTB and ACT presented with high rates of detectable +/++ fluorescence. Comparable fluorescence results were found for chondrosarcoma, osteosarcoma and Ewing’s sarcoma among bone and dedifferentiated liposarcoma, myxofibrosarcoma and undifferentiated pleomorphic sarcoma among soft tissue sarcomas. Because of these findings, further investigation of a possible incorporation of 5-ALA FGS in the clinical care of Orthopedic Oncology patients seems warranted. Taking the varying resection requirements and recommendations for different tumor entities into account, we propose two possible avenues for how 5-ALA FGS might positively affect surgical treatment outcomes. For locally aggressive tumors of bone, such as GCTB and ACT, the preferred treatment consists of curettage with the use of adjuvants and defect filling using bone cement or bone grafts [9–11]. Due to this intralesional treatment, a significant rate of local recurrences is observed [9–11]. Endoscopically assisted curettage of these tumors using 5-ALA PDD may be useful in discriminating between residual tumor and healthy tissue. Because of a more thorough and complete curettage, local control might improve. In contrast, the common goal in both bone and soft tissue sarcoma treatment is achieving a complete tumor resection with clear margins [12, 13]. In the proximity of major vessels and nerves, margins are often close and pathological workup might conclude microscopically contaminated margins despite a macroscopically clear aspect. Examination of both the resection specimen and the tumor bed for 5-ALA mediated tumor fluorescence might help detect such areas and facilitate targeted re-resections within the same operation.

Statistical analysis of a therapeutic effect of PDT on tumor tissues was not possible in this study due to a high rate of spontaneous tumor regression of xenografts inoculated on the CAM. In the absence of a functional chick immune system [16], insufficient laceration of CAM vessels and insufficient perfusion of the tumor xenograft, especially in areas not directly adjacent to the CAM, are the most likely causes for this phenomenon. In line with the findings of a high rate of spontaneous regression in this study, Sys et al. report a rate of partial necrosis in 15.9% and complete necrosis in 56.3% of samples in their 2012 study [21]. We did, however, observe a dose-dependent increase of chick death after PDT, which may help to adjust doses for future experiments. Improved tumor viability rates after application of primary sarcoma cell suspensions (*n* = 2) on the CAM suggests a suitability of sarcoma cell lines in determining tumor fluorescence (PDD) and the effect of PDT on musculoskeletal tumors. This observation may be used to confirm the findings of this study and gain further knowledge in a subsequent analysis.

While engraftment of a wide range of benign, borderline and malignant musculoskeletal tumors was observed in this study, the high rate of partial to complete regression of tumor samples and short investigation period limit the significance of findings and possible benefits of the CAM model using macroscopic tumor xenografts. In their review of available human sarcoma cell lines, Hattori et al. present a limited number of available sarcoma cell lines in comparison with other cancers [22]. In the absence of available human sarcoma cell lines, the possibility of investigating musculoskeletal donor tumors in their human microenvironment provided with nutrients by their chick host is a redeeming aspect of the experimental setup of this study, as some musculoskeletal tumors will not thrive in primary or established cell cultures.

## Conclusions

Despite a high rate of partial to complete regression of tumor samples, the CAM model proves to be an apt and promising non-rodent in vivo model for musculoskeletal tumors. A high rate of observed 5-ALA associated fluorescence of musculoskeletal tumors suggests its capability in enhancing tumor tissues against an in vivo tissue background and supports further investigation of its applicability to improve resection margins and decrease local recurrence rates for selected musculoskeletal tumors.

## Data Availability

The datasets used and/or analyzed in this study are available from the corresponding author on reasonable request.

## Abbreviations

FGS: Fluorescence-guided surgery
5-ALA: 5-Aminilevulinic acid
CAM: Chorio-allantoic membrane
PDT: Photodynamic therapy
ICG: Indocyanine green
MB: Methylene blue
AO: Acridine orange
PDD: Photodynamic detection
GCTB: Giant cell tumor of bone
ACT: Atypical chondrogenic tumor
CT: Computed tomography
MRI: Magnetic resonance imaging
PPIX: Protoporphyrin IX
